# Building the toolkit to address malaria resurgence and radical cure of vivax malaria in Ethiopia: a meeting report

**DOI:** 10.1186/s12936-026-06050-4

**Published:** 2026-07-31

**Authors:** Tamiru Shibiru Degaga, Heven Sime, Shazia Ruybal-Pesántez, Ashley Osborne, Muthoni Mwaura, Samuel Alemu Bamboro, Hellen Mnjala, Getachew Mekonnen, Saba Ermias, Anneleye Fantahun, Khor Puoch, Semira Abdelmenan, Miraf Mesfin, Yohannes Hailemichael, Endalamaw Gadisa, Dagmawi Hailu, Eyerusalem Beyene, Damtie Lankir, Gebregorgis Teklu, Hassen Mamo, Eyob Amare, Kebede Etana, Yonas Temesgen, Bisrat Nigusse, Wondimagegn Adissu, Asrat Hailu, Delenasaw Yewhalaw, Asnakew Kebede, Jihad Abanegash, Hiwot Teka, Sineshaw Legese, Asefaw Getachew, Bashir Abdi, Mulgeta Minale, Adugna Abera, Geremew Tasew, Ketema Tafess, Abay Sisay, Negash Seyoum, Dereje Dilu, Abdi Aliyi, Angela Devine, Ashenafi Assefa, Sarah Auburn, Kamala Thriemer

**Affiliations:** 1 College of Medicine & Health Sciences, Arba Minch University, Arba Minch, Ethiopia; 2 Global and Tropical Health Division, Menzies School of Health Research and Charles Darwin University, Darwin, Australia; 3 Ethiopian Public Health Institute, Addis Ababa, Ethiopia; 4 Barcelona Institute for Global Health (ISGlobal), Barcelona, Spain; 5 Department of Health Ethics and Society, Care and Public Health Research Institute (CAPHRI), Maastricht University, Maastricht, The Netherlands; 6 PATH Malaria Control and Elimination Partnership in Africa (MACEPA), Addis Ababa, Ethiopia; 7 PATH, Partnership for Vivax Elimination (PAVE), Addis Ababa, Ethiopia; 8 Gambella Regional Health Bureau, Gambella, Ethiopia; 9 Addis Continental Institute of Public Health, Addis Ababa, Ethiopia; 10 Malaria and Neglected Tropical Diseases Research Division, Armauer Hansen Research Institute, Addis Ababa, Ethiopia; 11 Amhara Regional Health Bureau, Bahir Dar, Ethiopia; 12 Aksum University, Aksum, Ethiopia; 13 Addis Ababa University, Addis Ababa, Ethiopia; 14 Tigray Regional Health Bureau, Mekelle, Ethiopia; 15 Diseases Prevention and Control Directorate, Ministry of Health, Addis Ababa, Ethiopia; 16 Jimma University, Jimma, Ethiopia; 17 Independent Consultant, Addis Ababa, Ethiopia; 18 Dire Dawa City Administration Health Bureau, Dire Dawa, Ethiopia; 19 Independent Consultant, Global Health and Development Solutions PLC, Addis Ababa, Ethiopia; 20 Somali Regional Health Bureau, Jigjiga, Ethiopia; 21 Benishangul-Gumuz Regional Health Bureau, Assosa, Ethiopia; 22 Institute of Pharmaceutical Sciences, Adama Science and Technology University, Adama, Ethiopia; 23 Harari Regional Health Bureau, Harar, Ethiopia; 24 Centre for Health Policy, Melbourne School of Population and Global Health, University of Melbourne, Melbourne, VIC Australia; 25 Institute for Global Health and Infectious Diseases, University of North Carolina at Chapel Hill, Chapel Hill, NC USA

## Abstract

Ethiopia has experienced a substantial resurgence in malaria in recent years, with increasing case numbers and a growing contribution of *Plasmodium vivax* in several regions. At the same time, new evidence supporting improved radical cure strategies, including high-dose primaquine and single-dose tafenoquine, alongside advances in molecular tools, offers opportunities to address persistent transmission and relapse. Against a backdrop of constrained funding and health system challenges, a stakeholder meeting was convened in Addis Ababa in November 2025 to review emerging evidence and discuss implications for malaria control and elimination in Ethiopia. The two-day meeting brought together 38 representatives from the National Malaria Control Programme, research institutions, regional health bureaus, and implementation partners. Key themes emerging from the meeting included: (i) systemic challenges shaping malaria control, including funding constraints, operational disruptions, and climate-related pressures; (ii) uncertainty around the contribution of relapse to overall *P. vivax* burden and the potential role of molecular tools to address this gap; (iii) important health system considerations for the introduction of G6PD-guided radical cure, particularly regarding service delivery levels and equity; (iv) the central role of economic evidence, with substantial uncertainty around drug pricing and implementation costs; and (v) the need for strengthened collaboration between researchers and policymakers to support evidence-informed decision-making. The meeting highlighted that, while more effective tools for *P. vivax* radical cure are available, their adoption in Ethiopia will depend on addressing key evidence gaps, particularly around relapse burden, feasibility, and cost-effectiveness. Given the current challenges, there is a pressing need for context specific research and continued dialogue between stakeholders, which will be critical to inform policy decisions and support progress towards malaria elimination.

**Supplementary Information:**

The online version contains supplementary material available at 10.1186/s12936-026-06050-4.

## Introduction

Ethiopia achieved significant progress in malaria control between 2005 and 2020 through scaled-up interventions including expanded diagnostics, the introduction of artemisinin-based combination therapies (ACTs) and the health extension program. Recently, however, the country has experienced a substantial resurgence in malaria, with national case numbers increasing from 900,000 recorded cases in 2019 to between 7.3 and 12 million cases in 2024 [1, 2]. An estimated 282 million malaria cases were reported globally in 2024 across 80 endemic countries—about 9 million more than in 2023. Increases were largely concentrated to a few settings, with Ethiopia alone accounting for roughly one-third of the global increase and, together with Madagascar and Yemen, represented 58% of the estimated rise from 2023 to 2024 [3]. Current evidence shows a reduction in cases compared to 2024 (supplementary Table 1 and supplementary Fig. 1), with *Plasmodium falciparum* accounting for the majority of infections. However, *P*. *vivax* remains a significant contributor to the burden of disease, and, in some regions, is even increasing in its contribution, reaching over 40% of cases in Amhara, > 30% in Tigray, and between 10 to 25% in Somali and Dire Dawa (personal communication regional health bureau representatives).

Ethiopia’s National Malaria Strategic Plan is organised around five pillars, each designed to guide the country toward the vision of a malaria-free Ethiopia [4]. These include (i) stratifying districts along the elimination continuum; (ii) intensifying programme approaches and interventions; (iii) strengthening surveillance as a core intervention; (iv) enhancing stewardship, accountability, and community and political engagement; and (v) accelerating the uptake of new tools and technologies [4]. Pillar 5 recognises that achieving elimination will require introducing innovations that address current gaps in diagnosis and treatment protocols, including tools that support more effective treatment of vivax malaria, as well as improvements in surveillance to better understand transmission foci, case importation, and the spread of parasite resistance to antimalarials and diagnostics. The strategic framework therefore provides the policy basis for considering new approaches.

Ethiopia currently uses a low dose primaquine treatment (3.5 mg/kg total dose) given over 14 days without G6PD testing to prevent relapsing malaria (referred to as radical cure). Over the past decade, a growing body of evidence has strengthened the case for more effective treatment for *P. vivax*, particularly high-total-dose primaquine regimens and single-dose tafenoquine [5–7]. Recent multi-country pooled analyses have demonstrated that a high total dose of primaquine (7 mg/kg) provides substantially greater protection against relapse compared with the low-dose (3.5 mg/kg total dose) regimen [8, 9], which has led to a WHO recommendation of the high-dose primaquine regimen (7 mg/kg total dose given over 7 or 14 days) [10]. Importantly, a country-specific analysis showed a clear benefit of the high-dose regimen within the Ethiopian context [11]. While 7-day regimens have been suggested as a means to improve adherence , evidence from the EFFORT trial indicates thateven with a shorter course, there remains a gap between efficacy and real-world effectiveness [12], with pill burden identified as a major contributor [13, 14].

In parallel, evidence for tafenoquine as a simplified, single-dose, anti-relapse treatment has continued to grow. Tafenoquine administered as a single 300 mg dose has demonstrated strong protection against recurrence in multiple settings, including in Ethiopia [6, 7, 12]. The one-day regimen offers clear operational advantages by removing adherence barriers inherent to multi-day primaquine courses, though, similar to high dose primaquine, the safety of tafenoquine depends on access to a semi-quantitative glucose-6-dehydrogenase (G6PD) testing. The EFFORT trial findings support the geographic extension of the current tafenoquine recommendation from the current focus on South America [10] to high burden areas such as Ethiopia [12].

While new radical cure options offer the potential to substantially reduce recurrent *P. vivax* malaria, their optimal deployment depends on a clearer understanding of the relative contribution of relapses to the overall disease burden. Molecular tools provide potential frameworks to disentangle relapse episodes from reinfections, thereby generating evidence to guide treatment policy and prioritisation. The increased availability of advanced sequencing platforms and improved genotyping tools provides new opportunities to strengthen malaria surveillance by enabling fine-scale characterization of malaria transmission and infection dynamics [15, 16]. Molecular data can complement routine epidemiological surveillance by supporting the ability to distinguish between imported cases and local infections [17, 18], which allows for monitoring cross-border spread [19, 20], and the emergence of markers associated with resistance to insecticides [21] antimalarial treatments and diagnostics [22–25]. These tools have already been applied for detailed investigation of localized outbreaks, helping to contextualize resurgence, importation, and residual transmission [26–28].

In the case of *P. vivax,* high-resolution molecular tools offer critical added value by improving classification of recurrent infections and supporting more accurate estimates of treatment efficacy in radical cure clinical trials [29, 30]. Microhaplotype genotyping of *P. vivax* cases has been successfully applied to cases from multiple radical cure trials in Ethiopia [12, 30]. In the EFFORT trial, genotyping of day 0 and recurrent *P. vivax* pairs revealed similar proportions of suspected relapses in each of the three treatment arms, although the absolute number of relapses remained higher in patients treated with low-dose primaquine compared to those treated with high-dose primaquine or single-dose tafenoquine [12]. In another trial, where individuals were treated with or without primaquine [31], genotyping demonstrated a lower frequency of suspected relapses in patients treated with primaquine compared to those without [30]. *P. vivax* genotyping samples from these trials provides important proof of concept for the ability of molecular surveillance to inform on relapse burden in not only clinical trials but also potentially broader surveillance frameworks to inform on drivers of resurgence and reservoirs of transmission before and after intervention changes [32]. However, critical questions pertaining to sampling, cost, and associated uncertainty behind the classification, as well as other potential implementation barriers, remain to be addressed.

The availability of more effective radical cure options alongside improved molecular tools comes at a critical moment for Ethiopia as the country faces an increasing malaria burden alongside significant funding constraints. Against this backdrop, the aim of the stakeholder meeting was to discuss experiences during the 2024 resurgence and beyond, as well as disseminate recent evidence on optimised radical cure strategies and molecular tools and discuss their potential implications for policy and practice in Ethiopia.

## Meeting description

A stakeholder meeting “Building the toolkit to address malaria resurgence and radical cure in Ethiopia” was held on the 28th and 29th November, 2025, in Addis Ababa and convened 38 representatives from the National Malaria Programme, Ethiopian research institutions, five regional malaria focal persons (Amhara, Tigray, Somali, Gambella and Dire Dawa), and implementation partners. The objective of the meeting was to share evidence relevant to the recent malaria resurgence and the potential impact of implementing *P. vivax* radical cure in Ethiopia, as well as discuss possible implications for national strategy and implementation planning.

The meeting was structured over two days. Day 1 focused on malaria priorities and resurgence, including clinical and molecular insights for both *P. falciparum* and *P. vivax.* This was followed by discussions on radical cure evidence, including primaquine dosing, effectiveness data for high-dose primaquine and single-dose tafenoquine, and the interpretation of recurrence patterns, as well as the operational and economic considerations for their implementation. The latter session was designed to inform future analysis plans and included an interactive question and answer session using Mentimeter (Table 1). The full agenda is provided in Supplementary Table 2.

**Table 1:** Questions asked to inform future health economic analyses

Question	Answer options
Do you want to see the cost results in ETB or USD?	ETB USD Both ETB & USD
What cost should we use for 7.5mg pill of primaquine?	US$0.16 (PMI) US$0.02 (pharmacy) Don't know Another cost
What cost should we use per dose of tafenoquine?	US$5.37 (Brazil) US$1.32 (PAVE Ethiopia) Don't know Another cost
Any other comments on what the drug prices should be?	[free text responses]
Is there anything we should change for G6PD screening costs?	[free text responses]
What costs should be included in the day 3 clinical review visit?	Consultation only Hb only Urine test only Both Hb & urine Day 3 review not needed
Which treatments require a day 3 clinical review visit?	7-day-high-dose PQ 14-day-low-dose PQ Tafenoquine No need for day 3 review
Any further comments on the costs or other things we should consider?	[free text responses]
Are there any other comparators that you are interested in for the cost-effectiveness analysis?	[free text responses]
Which perspective should we take for the analysis?	Healthcare provider Societal (healthcare provider + household costs)
Would it be helpful for us to explicitly model males testing as G6PD intermediate (30–69% activity)?	Yes No Not sure
What cost-effectiveness threshold do you think we should use for Ethiopia?	[free text responses]
Which outcome should we use for the primary analysis?	DALYs averted QALYs gained Malaria cases averted
Where would the funding for tafenoquine and G6PD screening come from if Ethiopia wanted to implement?	[free text responses]
Should we combine the EFFORT Ethiopia and Pakistan cost-effectiveness results?	Yes No Don't care
Any other questions or comments?	[free text responses]

DALY: disability-adjusted life-year; ETB: Ethiopian birr; PAVE: The Partnership for Vivax Elimination; PQ: primaquine; QALY: quality-adjusted life-year; TQ: tafenoquine; USD: United States dollar

Day 2 shifted from evidence summaries to practical application, with an emphasis on priority use cases for molecular tools to support routine decision-making. The national elimination approach focuses on district stratification, intensified interventions, and using surveillance as a core strategy through index case and foci investigation. Lessons learned from historically successful demonstration of malaria elimination in the Amhara region that drove down case burden through integrated high-quality data collection, advanced serological and molecular tools and strengthened core interventions were presented. Sessions were delivered as short presentations followed by a moderated round table discussion to capture stakeholder perspectives and define priority analyses, along with next steps (Supplementary Table 3).

Throughout the meeting, notes were taken and collated to present key discussion points. The following themes emerged from the discussion: (i) systemic challenges shaping the response in Ethiopia, (ii) molecular tools to understand the relative contribution of relapse and other use cases for molecular tools, (iii) health system considerations for the introduction of shorter more effective radical cure options, (iv) cost considerations and the role of economic evidence, (v) Research–policy collaboration.

## Systemic challenges shaping Ethiopia’s malaria response

Stakeholders noted that the most pressing challenge for malaria control and eventual elimination is the combination of a reduced funding landscape [3], climate change and operational disruptions [33]. Together, this compounded population displacement and weakened routine health services, putting further strain on supply chains and workforce capacity [33, 34].

These systemic pressures have likely contributed to the recent resurgence of malaria and limited the programme’s ability to sustain and advance interventions. As such, all technical considerations—whether related to radical cure, G6PD testing, molecular tools, or economic evidence—may be viewed within the constrained health-system and financial landscape. Prioritising feasible, affordable, and operationally realistic strategies will be essential for Ethiopia to control malaria and eventually progress toward malaria elimination.

## The need to understand the relative contribution of relapse to the overall case burden and other use cases for molecular tools

Participants reviewed the most recent efficacy and effectiveness data from the EFFORT study and other ongoing work in Ethiopia [11, 12, 35]. While these data sources continue to strengthen the case for improved *P. vivax* radical cure strategies, the malaria program does not yet prioritize the use of high-dose primaquine (7mg/kg total dose) or single-dose tafenoquine combined with G6PD testing. Reasons for this include concerns over the cost but also uncertainty about the relative contribution of relapses versus new infections to the overall case burden, as well as the difficulty in estimating the potential public-health impact of more effective radical cure regimens. Molecular tools to distinguish relapses from new infections were discussed as a key component to address this knowledge gap. Although such tools are progressing rapidly, they are not yet fully validated and remain confined to academic research. Further research is needed before guidelines can be established on the integration of molecular correction for *P. vivax* radical cure trials analogous to the WHO guidelines for PCR correction of recurrent *P. falciparum* infections in therapeutic efficacy surveys [36]. While the application of molecular recurrence classification focused primarily on clinical trials, the potential utility in other surveillance frameworks was also noted. For example, the contribution of relapsing infection in driving the resurgence of *P. vivax* in different areas of Ethiopia remains unclear. However, the necessity of paired “day 0” and recurrent infections brings requirements for new sampling strategies and associated logistical and cost implications, as well as analytical considerations. Research is expanding on sampling strategies for malaria molecular surveillance, but much of the focus has been on *P. falciparum* [37] while focused efforts that consider the unique biology of *P. vivax* are greatly needed.

A consideration regarding the cost-impact balance of molecular surveillance is its potential to support use cases beyond recurrence classification. For example, in the context of resurgence, there was growing interest in leveraging parasite molecular data to identify blood-stage drug resistance signals as well as determining the relatedness of the circulating lineages in the current infections. However, at present there are no validated molecular markers for *P. vivax* drug resistance including to chloroquine, the frontline treatment for this species in Ethiopia [38]. Molecular data may nevertheless help characterise parasite relatedness, long-distance importation and cross-border spread [39]. Clearer operational definitions and methods that integrate molecular data with epidemiological and modelling frameworks, will be critical to move from descriptive analyses to actionable outputs. Stakeholders agreed that ongoing methodological developments and locally generated molecular data will be critical to inform future policy decisions, build confidence in adopting optimised radical cure strategies, and support the operationalisation of malaria molecular surveillance.

## Health-system considerations for the introduction of shorter more effective radical cure options

The discussion highlighted substantial health-system considerations for the introduction of optimised radical cure regimens, particularly regarding where G6PD testing and treatment should be delivered. According to national policy, approximately 70% of malaria cases are expected to be diagnosed and treated at the health-post level, with only 30% managed in health centres. Under this model, implementing G6PD-guided high-dose primaquine (7 mg/kg total dose) or tafenoquine may face challenges, due to possible limitations around staffing, training, and infrastructure at health posts. Conversely, restricting implementation to health centres would reduce operational complexity but may limit population-level impact. Several participants noted that policy does not always reflect reality in some settings. In some areas, participants describe up to 70% of patients seeking care at health centres rather than health posts. Stakeholders agreed that a better understanding of actual care-seeking behaviour across different regions of Ethiopia is essential to inform an equitable and operationally realistic roll-out strategy.

## Cost considerations and the role of economic evidence

Economic evaluation was identified as central to informing policy decisions on radical cure. Since G6PD screening was included for all participants in the EFFORT trial [12], a model-based cost-effectiveness analysis was developed to compare new radical cure options against current practice in Ethiopia, where low-dose primaquine (3.5mg/kg total dose over 14 days) is prescribed without G6PD screening.

Drug prices emerged as one of the most influential parameters on the preliminary results, which suggested that tafenoquine could be cost-saving compared with high-dose primaquine under some assumed procurement prices. This relationship reversed under alternative price scenarios. Accordingly, much of the discussion was focused on trying to identify which costs would be most appropriate given the funding shifts that have occurred since the trial concluded. Participants highlighted substantial uncertainty around both primaquine and tafenoquine costs, with one participant commenting that, “*The prices of drug should be as low as possible. In our context, it would be nicer if they are given for free.”* These findings underscored the need for two-way sensitivity analyses to explore a range of plausible price combinations.

**Fig. 1:**
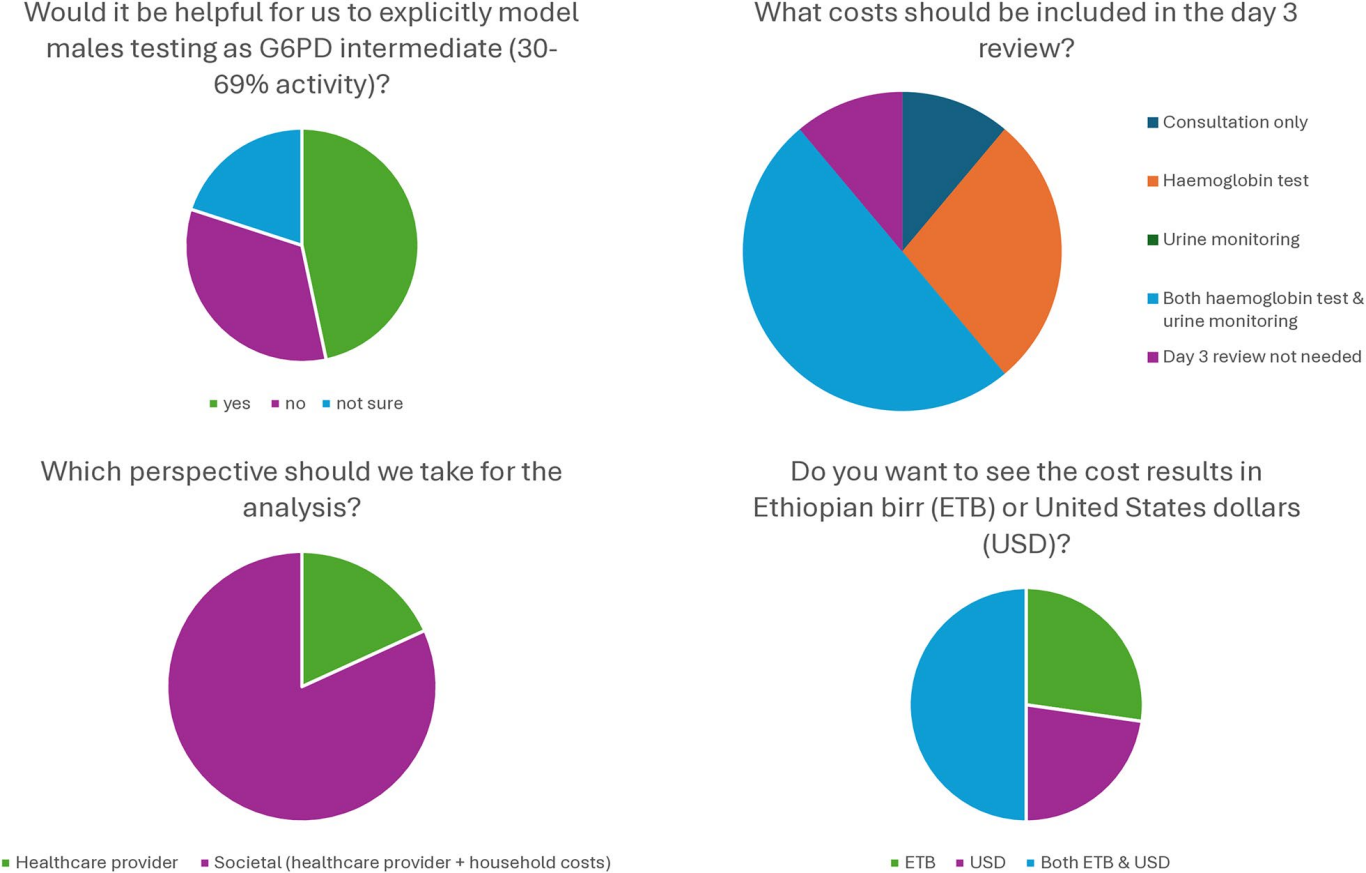
Selected results from the interactive health economics discussions

There was no clear consensus on whether patients with intermediate G6PD activity should be explicitly modelled, or on the extent to which follow-up visits (e.g., day 3 clinical review visit with haemoglobin and urine monitoring) should be incorporated for different regimens (Fig. 1). These decisions were acknowledged to have implications for both safety monitoring and overall costs.

Stakeholders expressed a strong preference for adopting a societal perspective that accounts for household-level costs in addition to health system expenditure (Fig. 1). While disability-adjusted life-years would facilitate comparison with other health programs, most participants favoured presenting cost per malaria case averted as the primary outcome (Fig. 1). Ethiopia currently lacks an explicit cost-effectiveness threshold, and uncertainty remains regarding how results would ultimately be interpreted for policy decisions.

## Research–policy collaboration and next steps

The meeting underscored the importance of strengthening collaboration between researchers, the National Malaria Programme, regional health bureaus, and implementing partners, to ensure that emerging evidence meaningfully informs policy and practice. Participants emphasised the importance of compiling and synthesising evidence across research groups to provide the National Malaria Programme with a clearer, more coherent, evidence base. Discussions highlighted that research findings and operational data are often generated across different institutions and projects in relative isolation, with limited opportunities for regular synthesis and dialogue with policymakers and programme stakeholders. A consolidated view of the available data—rather than isolated study outputs—will greatly enhance the programme’s ability to evaluate options, assess feasibility, and identify the most impactful pathways for reducing *P. vivax* burden.

This dialogue comes at a critical moment. Ethiopia is preparing for the next iteration of its national malaria strategic plan within the current funding landscape, which further elevates the importance of aligning research evidence with programmatic priorities, as a robust case for investment will require clear articulation of the expected impact, feasibility, and cost-effectiveness of proposed interventions. The meeting therefore provided an opportunity to strengthen communication between researchers and policymakers around emerging evidence and future priorities. Overall, success in malaria control and elimination will depend on strengthening health systems, enhancing surveillance and response, generating evidence for integration of tailored new tools and ensuring sustained political and financial commitment.

## Supplementary Information

Additional file1

## Data Availability

No datasets were generated or analysed during the current study.

## Abbreviations

ACT: Artemisinin combination therapies
G6PD: Glucose-6-phosphate dehydrogenase
