# Supplementary material for: Building the toolkit to address malaria resurgence and radical cure of vivax malaria in Ethiopia: a meeting report
Source: Malar J. 2026 Jul 31;25:278. doi: 10.1186/s12936-026-06050-4 (PMC13425933; doi:10.1186/s12936-026-06050-4)
Supplement: Supplementary file 1 — Additional file1 [file 12936_2026_6050_MOESM1_ESM.docx]

**Supplementary Table 1:** **Malaria case data by region**.

Data was obtained from the Federal Ministry of Health (FMoH), which has authorized access to the DHIS2 platform. The data were provided to HS upon our request after informing the Ministry that they would be used to supplement the analyses presented in this publication.

| **Region** | **2021** | **2022** | **2023** | **2024** | **2025** |
| --- | --- | --- | --- | --- | --- |
| Addis Ababa | 2,014 | 3,397 | 6,296 | 19,266 | 18075 |
| Afar | 88204 | 107209 | 133446 | 146058 | 149433 |
| Amhara | 498189 | 840796 | 1098853 | 1718560 | 1438486 |
| Benishangul-Gumuz | 99710 | 167781 | 224908 | 386116 | 478607 |
| Central Ethiopia | 66001 | 138227 | 135924 | 477738 | 483678 |
| Dire Dawa | 934 | 8884 | 11382 | 25052 | 20449 |
| Gambella | 73582 | 143408 | 144272 | 161044 | 148396 |
| Harari | 872 | 689 | 481 | 7932 | 5145 |
| Oromia | 143954 | 414641 | 1285422 | 4318198 | 2538318 |
| Sidama | 31957 | 130046 | 139653 | 248485 | 203910 |
| Somali | 109307 | 89928 | 62183 | 54590 | 61335 |
| South Ethiopia | 214461 | 292681 | 327394 | 820648 | 1076194 |
| South West Ethiopia | 88487 | 287366 | 556832 | 1187614 | 1105120 |
| Tigray* | 9435 | 70146 | 301849 | 384586 | 215824 |
| **Total** | **1,427,107** | **2,695,199** | **4,428,895** | **9,955,887** | **7,942,970** |

*For the Tigray Region, data from the Tigray Regional Health Profile for 2021 to 2024. Data for 2025 was supplemented by the FMoH.

**Supplementary Figure 1: Annual malaria case data from Ethiopia between 2021 and 2025**. (A) Annual malaria cases from 2021 to 2025 in Ethiopia. (B) Malaria cases by region in Ethiopia between 2021 and 2025.

**
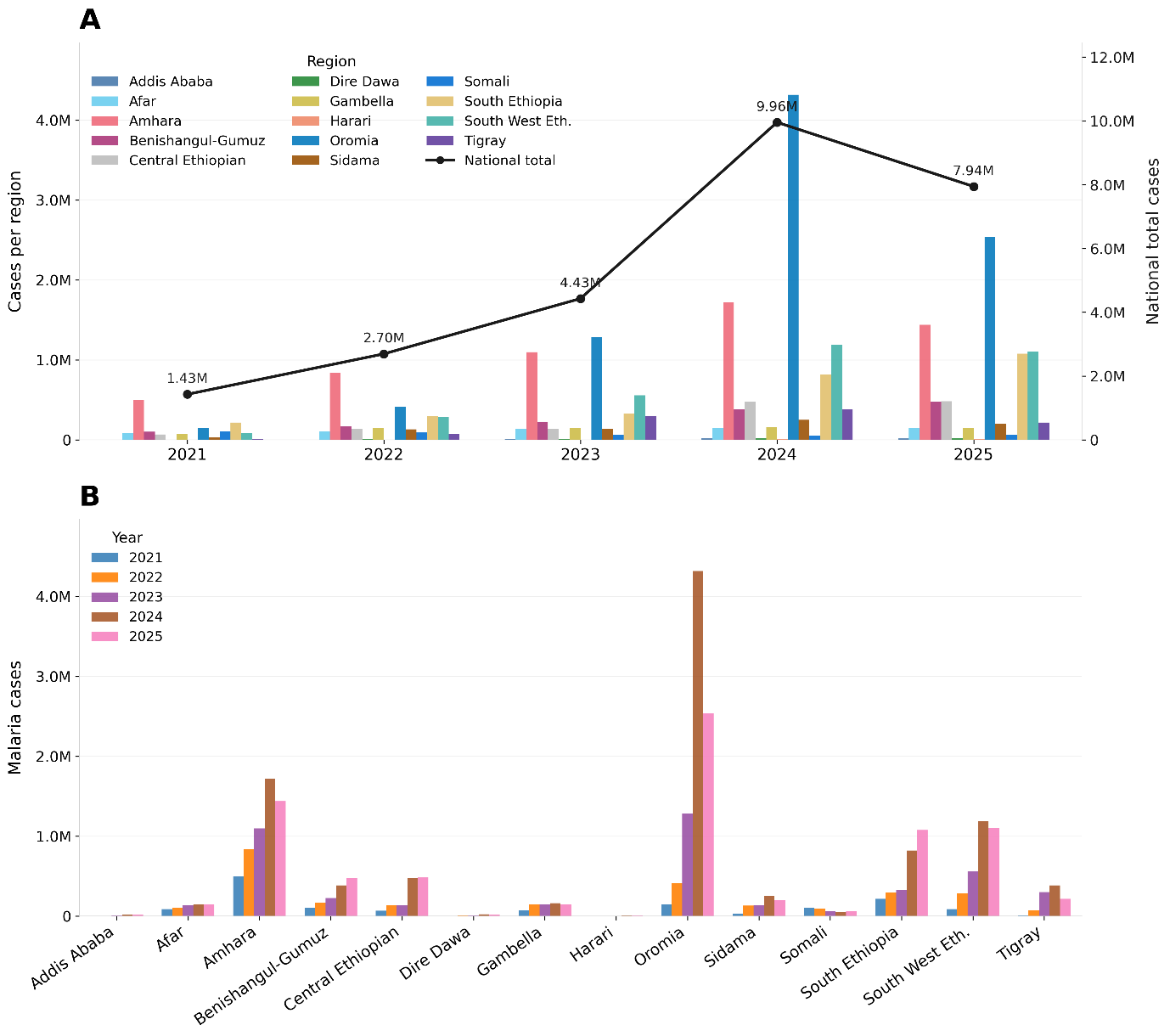
**

**Supplementary table 2: Agenda Stakeholder Meeting on Malaria: Building the Toolkit to Address Resurgence and Radical Cure (Day 1)**

| **Stakeholder meeting on malaria: building the toolkit to address resurgence and radical cure** | | | |
| --- | --- | --- | --- |
| **Nov 28^th^ 2025** | | | |
| Time | Duration | Title | Presenter |
| 09:00-09:15 | 15 min | Introductions/overview of agenda | Ashenafi Assefa & Tamiru Degaga |
| 09:15-09:25 | 20min | Overview of Ethiopia’s current malaria challenges/priorities | Kebede Etana |
| 09:25-09:45 | 20 min | Resurgence of P. falciparum – clinical and molecular data insights and recommendations | Ashenafi Assefa |
| 09:45-10:00 | 15 min | Resurgence of P. vivax - clinical and molecular data insights and recommendations | Heven Sime |
| 10:00-10:30 | 30 min | Discussion |  |
| 10:30-11:00 | 30 min | Tea break |  |
| 11:00-11:20 | 20 min | Radical cure evidence - primaquine optimal dose for Ethiopia | Tamiru Degaga |
| 11:20-11:30 | 10 min | Discussion |  |
| 11:30-11:50 | 20 min | Radical cure evidence – effectiveness of high dose primaquine and tafenoquine, results from the EFFORT study | Kamala Thriemer |
| 11:50-12:00 | 10 min | Update on PAVE study | Endalamaw Gadisa |
| 12:00-12:20 | 20 min | Discussion |  |
| 12:20-12:40 | 20 min | Radical cure evidence – not all recurrences are reinfections | Ashley Osborne and Shazia Ruybal-Pesántez |
| 12:40-12:50 | 10 min | Questions/Discussion |  |
| 12:50-14:00 | 1.10 hour | Lunch break |  |
| 14:00-14:20 | 20 min | Acceptability of high dose primaquine and tafenoquine – what matters for implementation | Muthoni Mwaura |
| 14:20-14:40 | 20 min | Discussion |  |
| 14:40-15:10 |  | Cost effectiveness of radical cure | Angela Devine |
| 15:10-15:30 | 20 min | Discussion |  |
| 15:30-15:45 | 15 min | Meeting Closure and reception |  |

**Supplementary table 3: Agenda Stakeholder Meeting on Malaria: Building the Toolkit to Address Resurgence and Radical Cure (Day 2; Draft and Dynamic)**

| **Stakeholder meeting on malaria: building the toolkit to address resurgence and radical cure** | | | |
| --- | --- | --- | --- |
| **Nov 29^th^ 2025** | | | |
| **Aims**  • Define programmatic and operational activities related to outbreak/resurgence and elimination  • Discuss priority research and operational questions  • Define sampling, data, and analysis requirements | | | |
| Time | Duration | Title | Presenter(s) |
| 09:00–09:15 | 15 min | Introduction and overview of Day 2 agenda | Ashenafi Asefa Shazia Ruybal-Pesántez |
| 09:15–10:15 | 10 min each | Situation reports from malaria focals (with emphasis on *P. vivax*) | Regional focal representatives |
| 10:15-10:35 | 20 min | General discussion |  |
| 10:35-11:00 |  | Tea break |  |
| 11:00-11:20 | 20 min | Sub national malaria elimination effort in Ethiopia | Dereje Dilu |
| 11:20-11:40 | 20 min | Malaria elimination experiences and lessons: Amhara region example | Asfaw Getachew |
| 11:40-11:50 | 10 min | Cost effectiveness of MMS and implications | Angela Devine |
| 11:50-12:10 | 20 min | Discussion |  |
| 12:10-13:30 | 1 hr 20 min | Lunch break |  |
| 13:30-13:50 | 20 min | Use cases and sampling for malaria molecular surveillance | Shazia Ruybal-Pesántez |
|  |  | Breakout group organization | Ashenafi Assefa |
| 13:50–15:00 | 1 hr 10 min | Round-table discussions (4 tables):  **Programmatic & Operational Questions for malaria elimination in Ethiopia**  1. What are the current operational bottlenecks affecting malaria control and elimination activities in Ethiopia?  2. What evidence is needed to support shifting from control to *elimination* strategies?  3. What are the gaps in implementing radical cure for *P. vivax*?  Elective Questions (choose one or two questions to discuss)  1. Genetic/ genomic evidence for lineage expansion, drug resistance markers  2. Are there early indicators of drug or diagnostic failure?  3. Is MDA an option for malaria elimination Ethiopia  4. What lessons can Ethiopia adapt from elimination programs in Asia and Latin America?  5. What is required to establish and maintain malaria-free zones?  6. Region-specific outbreak reports and lessons?  7. Mapping of high-risk populations: migrants, agricultural workers, border communities  8. Any other thoughts or relevant questions | Table groups |
| 15:00–16:00 | 1 hr | Group presentations | Group leaders |
| 16:00-16:15 | 15 min | Discussion and wrap-up |  |
|  |  | Program end |  |
